# A cascade nanosystem with “Triple-Linkage” effect for enhanced photothermal and activatable metal ion therapy for hepatocellular carcinoma

**DOI:** 10.1186/s12951-024-02551-z

**Published:** 2024-06-14

**Authors:** Shuo Yu, Huan Shen, Xi Chen, Hong Wang, Chenyang He, Tinghua Hu, Gang Cao, Lu Zhang

**Affiliations:** 1 Department of General Surgery, The Second Affiliated Hospital, Xi’an Jiaotong University, Xi’an, 710000 China; 2 Department of Tumor and Immunology in Precision Medical Institute, The Second Affiliated Hospital, Xi’an Jiaotong University, Xi’an, 710000 P. R. China; 3 The Breast Disease Diagnosis and Treatment Center, The Second Affiliated Hospital, Xi’an Jiaotong University, Xi’an, 710000 P. R. China; 4 Department of Respiratory and Critical Care Medicine, The First Affiliated Hospital, Xi’an Jiaotong University, Xi’an, 710000 P. R. China

**Keywords:** Hepatocellular carcinoma, Triple-linkage effect, Energy metabolism, Enhanced photothermal therapy, Activatable metal ion therapy

## Abstract

Due to the limitations of single-model tumor therapeutic strategies, multimodal combination therapy have become a more favorable option to enhance efficacy by compensating for its deficiencies. However, in nanomaterial-based multimodal therapeutics for tumors, exploiting synergistic interactions and cascade relationships of materials to achieve more effective treatments is still a great challenge. Based on this, we constructed a nanoplatform with a “triple-linkage” effect by cleverly integrating polydopamine (PDA), silver nanoparticles (AgNPs), and glucose oxidase (GOx) to realize enhanced photothermal therapy (PTT) and activatable metal ion therapy (MIT) for hepatocellular carcinoma (HCC) treatment. First, the non-radiative conversion of PDA under light conditions was enhanced by AgNPs, which directly enhanced the photothermal conversion efficiency of PDA. In addition, GOx reduced the synthesis of cellular heat shock proteins by interfering with cellular energy metabolism, thereby enhancing cellular sensitivity to PTT. On the other hand, H_2_O_2_, a by-product of GOx-catalyzed glucose, could be used as an activation source to activate non-toxic AgNPs to release cytotoxic Ag^+^, achieving activatable Ag^+^-mediated MIT. In conclusion, this nanosystem achieved efficient PTT and MIT for HCC by exploiting the cascade effect among PDA, AgNPs, and GOx, providing a novel idea for the design of multimodal tumor therapeutic systems with cascade regulation.

**Graphical abstract:**

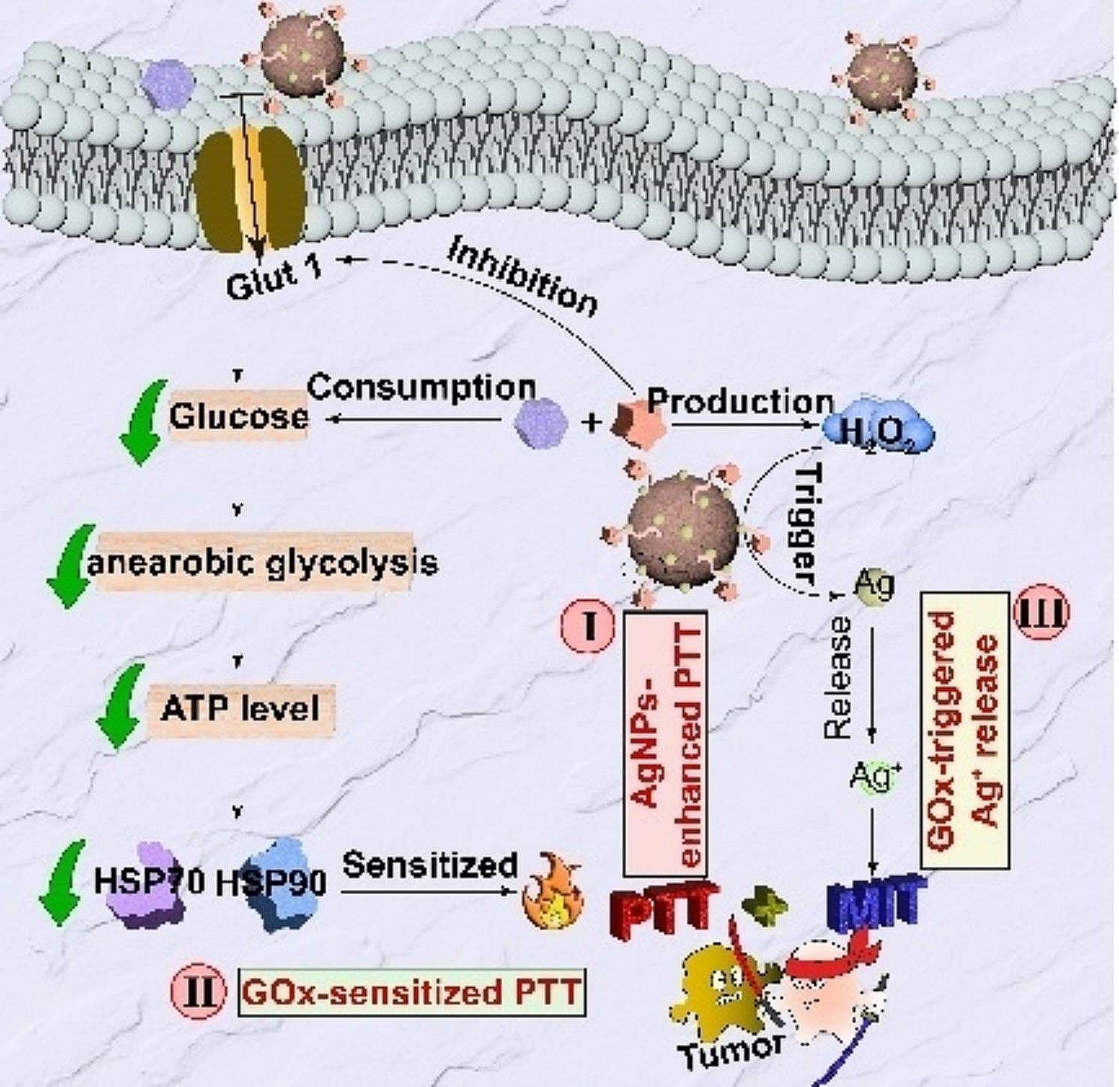

**Supplementary Information:**

The online version contains supplementary material available at 10.1186/s12951-024-02551-z.

## Introduction

Hepatocellular carcinoma (HCC) is the most predominant type of liver cancer [1, 2]. Common treatments like surgical resection, transarterial chemoembolization (TACE), liver transplantation, transarterial radioembolization (TARE), and chemotherapy are effective to some extent but still have their drawbacks [3, 4]. For example, severe toxic side effects after chemotherapy and rejection reactions after liver transplantation make the treatment of HCC still extremely challenging [5, 6].

The exceptional structural and physicochemical characteristics of nanomaterials confer upon them superior lesion-site targeting capabilities and therapeutic functions [7, 8]. Consequently, they have garnered significant attention and utilization within the realm of tumor therapy in recent years. Novel therapeutic approaches rooted in nanomedicine, such as targeted chemotherapy [9], photodynamic therapy (PDT), photothermal therapy (PTT) [10], chemodynamic therapy (CDT) [11, 12], and immunotherapy [13], have been documented to substantially enhance the efficacy of HCC treatment while mitigating adverse effects. As one of the non-invasive thermal ablation therapies, PTT utilizes photothermal agents (PTAs) to convert light energy into heat energy for tumor destruction when exposed to an external light source, which has shown superiority over other therapies due to its low adverse effects and high specificity [14–16].

The efficacy of the PTT process is largely dependent on the PTAs utilized. Commonly employed PTAs include inorganic materials like gold nanoparticles, palladium nanosheets, and transition metal dichloride, as well as organic nanoparticles such as polypyrrole, polyaniline, and polydopamine (PDA) [17–20]. PDA is considered a promising PTA due to its ease of synthesis, good photostability, and excellent biosafety [21–23]. However, like other organic PTAs, the incomplete non-radiative conversion of PDA results in low efficiency of photothermal conversion under near-infrared light irradiation [24, 25]. Therefore, additional efforts are required to enhance the photothermal conversion efficiency of PTAs.

Metal-ion therapy (MIT) is a recognized method for treating tumors by manipulating intracellular metal ion levels to inhibit tumor growth or modulate immune responses [26]. Despite its potential, MIT is hindered by limitations such as poor specificity and inadequate changes in ion concentrations [27]. Nanomedicine technology has emerged as a promising strategy to enhance the delivery of metal ions and maintain optimal intracellular levels, thereby improving the efficacy of MIT [28]. Research in the field has shown that the inclusion of metal nanoparticles can enhance the photothermal conversion efficiency of photothermal agents through improved non-radiative transformation and enhanced charge transfer efficiency [25, 29]. Among metal nanoparticles, silver nanoparticles (AgNPs) are preferred by researchers for their biodegradability and favorable biosafety profile [30, 31]. In addition to augmenting photothermal therapy efficiency, AgNPs can release Ag^+^ ions, which are cytotoxic, from inert nanoparticles under oxidizing conditions, leading to cellular damage [32, 33].

Nevertheless, the efficacy of treatment is hindered by the limited optimism of the treatment effect, as the robust self-protection mechanisms of tumor cells significantly diminish their sensitivity to treatment [34–36]. For example, heat shock proteins (HSPs) are stress-responsive proteins found in mammals that are produced in large quantities to protect proteins from damage caused by heat stress [35, 37]. The increased expression of HSPs in cancer cells can reduce the efficacy of PTT. Therefore, the inhibition of HSP synthesis is a preferable strategy to overcome resistance in cancer cells. However, existing small molecule inhibitors of HSPs are limited by poor water solubility and high toxicity, while siRNAs are hindered by issues such as instability and degradation [38–40]. Consequently, there is an urgent need for a more effective approach to target HSP synthesis.

**Scheme 1:**
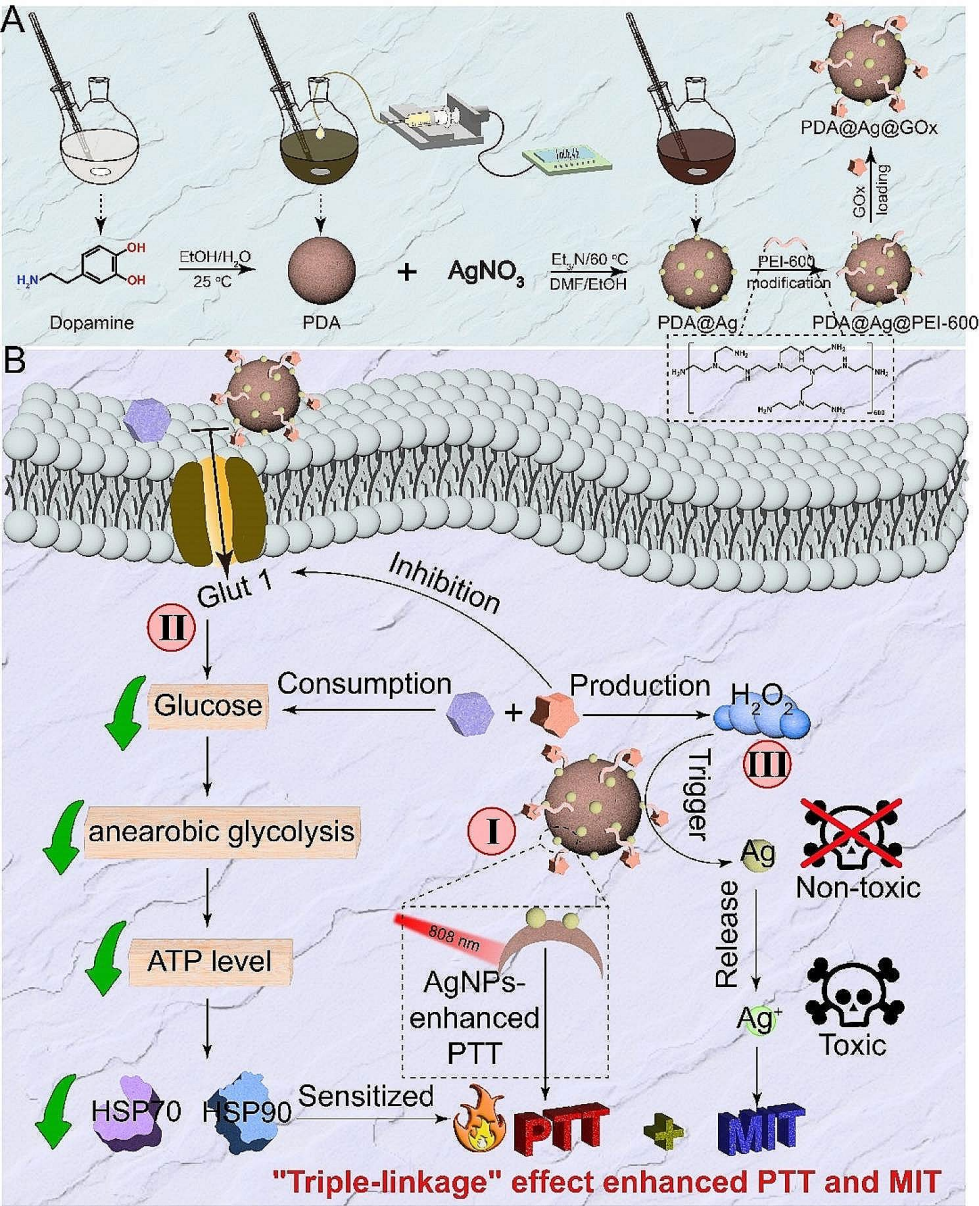
“Triple-linkage” effect nanoplatform for HCC treatment. (**A**) Schematic diagram of the synthesis of PDA@Ag@GOx. (**B**) Illustration of PDA@Ag@GOx for HCC treatment with enhanced PTT and MIT via “triple-linkage” effect: (I) AgNPs enhanced PTT effect, (II) GOx-sensitized PTT, and (III) GOx-triggered Ag^+^ release

Recently, glucose oxidase (GOx) has emerged as a promising candidate in the realm of tumor diagnosis and treatment [41–43], with our group having demonstrated its ability to disrupt HSPs synthesis by perturbing cellular glucose metabolism and diminishing intracellular energy production. Furthermore, the production of H_2_O_2_ as a by-product during the GOx-mediated catalytic process has been shown to elevate the oxidation state of cells [44–46], a factor that is advantageous for activatable MIT treatment. Based on this, we employed PDA as the core for the in situ synthesis of AgNPs on its surface, followed by the loading of GOx to construct a “triple-linkage” nanoplatform (PDA@Ag@GOx) for enhanced PTT and activatable MIT of HCC (Scheme 1A). Initially, the combination of PDA@Ag@GOx demonstrated improved PTT efficacy compared to PDA alone when exposed to NIR light, attributed to the enhanced non-radiative conversion facilitated by AgNPs decoration. Additionally, the presence of GOx further augmented the PTT effect by disrupting glucose metabolism in tumor cells, leading to reduced ATP production and subsequent inhibition of HSPs synthesis, resulting in a dual enhancement of PTT [47]. Moreover, the GOx-mediated generation of H_2_O_2_ could serve as a trigger for activating dormant AgNPs to release cytotoxic Ag^+^, thereby enabling Ag^+^-mediated MIT (Scheme 1B). Collectively, this nanosystem has potential to achieve highly efficient PTT and MIT for HCC through the rational integration of PDA, AgNPs, and GOx, leveraging the synergistic effects among these components, which provide a promising approach for the design of a tumor cascade regulation therapeutic system.

## Materials and methods

### Materials

Dopamine hydrochloride, AgNO_3,_ and glucose were purchased from Aladdin-Reagent Co. Ltd. (China). Glucose Oxidase (GOx), JC-1 probe, Hydrogen Peroxide Assay Kit, Calcein-AM and Enhanced BCA Protein Assay Kit, and Enhanced ATP Assay Kit were obtained from Beyotime Institute of Biotechnology (China). The Reactive Oxygen Species probe Dihydroethidium (DHE) was purchased from Applygen. All other materials were used directly as received unless otherwise specified.

### Instruments

TEM image was obtained on FEI-Tecnai G2 F20 at 200 kV. The hydrodynamic size and zeta potential were performed by dynamic light scattering (DLS) on a Litesizer 500 instrument. The UV − vis absorbance was measured by UV − vis spectroscopy (Lambda Bio40). The in vivo imaging experiments were conducted using IVIS imaging systems (PerkinElmer). Released Ag^+^ was collected by ICP-AES (Agilent 5110).

### Synthesis of PDA@Ag@GOx nanostructures

#### PDA synthesis

First, 2 mL NH_3_·H_2_O, 90 ml pure water, and 40 mL ethanol were mixed and stirred for 30 min, then 10 mL 50 mg/mL dopamine hydrochloride solution was added to stir for another 24 h. Subsequently, the mixture was centrifuged (12,000 rpm, 15 min) and washed with water three times to obtain the pure PDA nanoparticles.

#### PDA@Ag synthesis

PDA@Ag was prepared by reducing AgNO_3_ in situ on PDA. In brief, the PDA nanoparticles (6 mg) were dispersed in DMF (20 mL) containing TEA (50 µL) under stirring at 60 °C for 10 min. Subsequently, 4 mL ethanol solution of AgNO_3_ (3 mg/mL) was dropped into the mixture at the feeding rate of 1 mL/h. After that, the precipitation was centrifuged (12,000 rpm, 15 min) and washed with water for 3 times, and finally stored in water for later use.

#### PDA@Ag@Gox

150 µL PEI (100 mg/mL) was added to 3 mL PDA@Ag solution (1 mg/mL) to stir for 2 h, then the precipitate was collected and washed to obtain PDA@Ag@PEI, and dispersed in 3 mL of pure water. Then, 300 µL GOx (10 mg/mL) was added to the above PDA@Ag@PEI solution to stir at 4 ^o^C for 2 h. Finally, PDA@Ag@GOx was collected by centrifugation. PDA@GOx was synthesized in the same way as PDA@Ag@GOx except that PDA@Ag was replaced by PDA.

### H_2_O_2_ production

H_2_O_2_ production by PDA@Ag@GOx catalyzes glucose was tested using the Hydrogen Peroxide Assay Kit. PDA@Ag@GOx and PDA@Ag (100 µg/mL) were dispersed in 1 mg/mL glucose solution respectively, and the supernatant was collected via centrifugation at a given time point (0, 0.5, 1, 2, 4 h). Then the H_2_O_2_ concentrations in all samples were tested according to instructions of the Hydrogen Peroxide Assay Kit, and each sample was retested three times.

### Glucose detection

The ability of PDA@Ag@GOx to consume glucose was tested by using the DNS method. For the solution experiment, 0.1 mg PDA@Ag@GOx was added to 1 mL glucose solution for reaction, and then the glucose content at different time points was detected by the DNS reagent. Specifically, 1 mL reaction solution was mixed with 1 mL water and 3 mL DNS reagent, then the mixture was incubated at 100 ^o^C for 5 min. Finally, the UV-Vis absorption at 532 nm of the mixture was detected. The detection method at the cell level was consistent with that in the liquid phase experiment. For the cell experiment, the culture medium of cells treated with different samples was collected and used the same method to detect the ability of materials to consume glucose at the cellular level.

### pH change detection

100 µg/mL PDA@Ag@GOx was added in PBS (pH = 7.4) containing glucose (1 mg/mL), and the pH values at different time intervals were recorded by a pH meter.

### Ag^+^ release

PDA@Ag@GOx and PDA@Ag were dispersed in glucose solution (1 mg/mL) respectively, and placed in a rotary mixer for different times (0,4,8,12 h) at room temperature. Then, the PDA@Ag@GOx and PDA@Ag solutions incubated for different times were detected by full spectrum UV-Vis absorption to observe the change of silver absorption peak.

PDA@Ag@GOx and PDA@Ag were dispersed in glucose solution (1 mg/mL) respectively. After incubation at room temperature for 24 h, the supernatant was obtained by centrifugation and the content of Ag^+^ was detected by inductively coupled plasma emission spectrometer (ICP).

### Photothermal efficiency calculation

0.3 mL solution containing water and PDA, PDA@Ag, and PDA@Ag@GOx (at equal PDA concentration of 0.1 mg/mL) were separated into clear plastic centrifuge tubes. Then the solutions were exposed to 808 nm light irradiation at 0.8–1.0 W/cm^2^ for 5 min, and imaged by an IR thermal camera. Further, the temperatures at each time points were quantified by Guide software. Besides, five cycles of heating and cooling were also recorded to evaluate the photothermal stability of nanoparticles.

The photothermal conversion efficiency (*η*) was calculated according to classical method reported in the literature by following Equation:

$$\eta = \frac{hS\left({T}_{max}-{T}_{sur}\right)-{Q}_{in}}{I(1-{10}^{-A808})}$$

where *T*_*max*_ and *T*_*sur*_ stands for the maximum system temperature and the environmental temperature, respectively. *Q*_*in*_ is the heat dissipation from the light absorbed by the used tubes was determined as 20.3 mW. *I* is the input laser power (1.0 W/cm^2^) and A808 is the absorbance of nanoparticles at 808 nm. Subsequently, the *hS* value can be derived by following Equation:

$${\tau }_{s}= \frac{{m}_{D}{C}_{D}}{hS}$$

where *m*_*D*_ and *C*_*D*_ are the mass (0.3 mg) and heat capacity (4.2 J/g °C) of the solvent of deionized water, respectively. And τ_s_ is the time constant of the sample system, which can be calculated as following Equation:

$$t= -{{\tau }}_{s}\text{l}\text{n}\left({\theta }\right)$$

Here, θ can be expressed as following Equation:

$$\theta = \frac{T-{T}_{sur}}{{T}_{max}-{T}_{sur}}$$

In summary, the photothermal conversion efficiency (*η*) of PDA, PDA@Ag, and PDA@Ag@GOx could be calculated based on the above Equations step by step.

### Cell Culture

Mouse hepatoma cells (Hepa 1–6) were cultured in DMEM medium with 10% fetal bovine serum (FBS), 1% antibiotics (penicillin-streptomycin, 10,000 U/mL) at 37 °C in a humidified atmosphere with 5% CO_2_.

### Cytotoxicity test

The cytotoxicity of PDA@Ag@GOx against Hepa 1–6 cells was tested via MTT assay. Hepa 1–6 cells were placed in a 96-well plate and cultured for 24 h. Then the old medium was replaced by fresh medium containing different samples with concentration gradients, and cultured for another 4 h. After that, the groups treated with light were irradiated by 808 nm laser (1 W/cm^2^, 3 min), and the others were cultured in the dark all the time. After the cells were cultured for 24 h, MTT was added to each well and the cells were further cultured for 4 h. Then the medium was replaced by 150 µL DMSO to dissolve formazan, and the plate was measured by microplate reader at 570 nm. Then the cell viability (%) was calculated as (OD570(samples)/OD570(control)) X 100%.

### Live/dead cell staining analysis

The death of Hepa 1–6 cells caused by PDA@Ag@GOx was evaluated intuitively by the live/dead cells staining experiment. Hepa 1–6 cells were placed in a 96-well plate and cultured for 24 h. Then the old medium was replaced with fresh medium containing different samples and cultured for another 4 h. After that, the groups treated with light were irradiated by 808 nm laser (1 W/cm^2^, 3 min), and the others were cultured in the dark all the time. After 24 h incubation, all the cells were double stained with Calcein AM (labeled live cells) and PI (labeled dead cells) for 20 min, and finally observed by an inverted fluorescence microscope.

### JC-1 analysis

The JC-1 assay was conducted to study the mechanism of cell death. Hepa 1–6 cells were placed in a 6-well plate cultured for 24 h. Then the cells were treated with various samples. After treatment, the cells were stained with JC-1 for 15 min and further observed by an inverted fluorescence microscope.

### Intracellular ATP detection

The intracellular ATP level of Hepa 1–6 after various treatments was detected by the ATP Assay Kit. Heap 1–6 cells were placed in a 6-well plate and cultured for 24 h. Further, the cells were treated with PBS, GOx, PDA, PDA@Ag, and PDA@Ag@GOx respectively to co-culture for 24 h. Subsequently, the cells were lysed and centrifuged (12,000 g, 4 ^o^C, 5 min) to collect the supernatant after being washed with PBS. Finally, the ATP content in the supernatant was detected by the ATP Assay Kit.

### Western blot analysis

Hepa 1–6 were placed in 6-well plates and incubated for 24 h. Then the cells were undergone various treatments: (1) PBS-L, (2) PBS + L, (3) PDA@Ag-L, (4) GOx, (5) PDA@Ag@GOx-L, (6) PDA@Ag@GOx + L. The condition of light irradiation was 1 W/cm^2^, 3 min. After various treatments, the proteins were extracted from the treated cells. Then the extracted proteins were quantified, followed by electrophoresis and transmembrane. Subsequently, the PVDF membrane was incubated with Glut1 (Rabbit, R380464, ZEN-BIOSCIENCE, Cheng Du, CHINA), HSP70 (Rabbit, R24633, ZEN-BIOSCIENCE, Cheng Du, CHINA), HSP90 (Rabbit, R24635, ZEN-BIOSCIENCE, Cheng Du, CHINA) or β-actin (Rabbit, R23613, ZEN-BIOSCIENCE, Cheng Du, CHINA) primary antibody at 4 ^o^C overnight after protein blocking. Then the membrane was washed with TBST 3 times and followed by incubation with a secondary antibody at 37 ^o^C for 1 h. Finally, the specific protein expression was graphed by an omnipotent imager.

### LDH assay

The cytotoxicity of PDA@Ag@GOx was further detected by the LDH Cytotoxicity Assay Kit. Hepa 1–6 cells were placed in a 6-well plate and cultured for 24 h. Further, after the cells were cultured for 24 h after various treatments, the LDH released was measured according to the instructions of the LDH Assay Kit.

### In vivo tumor-targeting study

All live animal experiments were conducted according to the protocols of the Institutional Animal Care and Use Committee of the Animal Experiment Center of Xi’an Jiaotong University (Xi’an, China).

Hepa 1–6 cells were injected subcutaneously into male C57 mice to construct the tumor-bearing model. When the tumor volume reached 150 mm^3^, PBS, PDA, or PDA@Ag@GOx were intravenously injected into tumor-bearing mice respectively. A near-infrared thermal imaging system was used to record the thermal imaging pictures under irradiation with 808 nm laser (1 W/cm^2^, 3 min). The temperature of tumor tissue at different time points was quantified by Guide software.

### In vivo antitumor effect

The mice were randomly divided into 8 groups (6 mice per group) when the tumor volume reached 100 mm^3^. Then the mice were treated with (1) PBS, (2) PBS + L, (3) GOx, (4) PDA + L, (5) PDA@Ag-L, (6) PDA@Ag + L, (7) PDA@Ag@GOx-L, (8) PDA@Ag@GOx + L. Mice in the group requiring laser irradiation were irradiated with 808 nm (1 W/cm^2^, 3 min) after 24 h of injection. After that, the mice’s body weight and tumor volume were recorded and measured every day. Tumor volume (V) was calculated as width^2^ X length/2, and relative tumor volume (%) was defined as V/V_0_, where V_0_ was the tumor volume on the first day. After treatment, all the mice were sacrificed, and the tumor tissues and other major organs were collected to further evaluate the antitumor mechanism.

### Hemolysis test

We collected red blood cells (RBCs) from the peripheral blood of mice and incubated a series of concentrations of PDA@Ag@GOx with RBCs at 37 ^o^C for 30 min. After that, the samples were centrifuged to detect the absorption of the supernatant at 510 nm to calculate the hemolysis rate. RBCs treated with PBS acted as negative control and those treated with water acted as positive control.

### ALT&AST level detection

PDA@Ag@GOx was injected intravenously into mice for three consecutive days, after which the mice were executed to obtain peripheral blood, and the levels of AST and ALT were measured using AST and ALT assay kits, respectively.

## Results and discussion

### Preparation and characterization of PDA@Ag@GOx

**Fig. 1:**
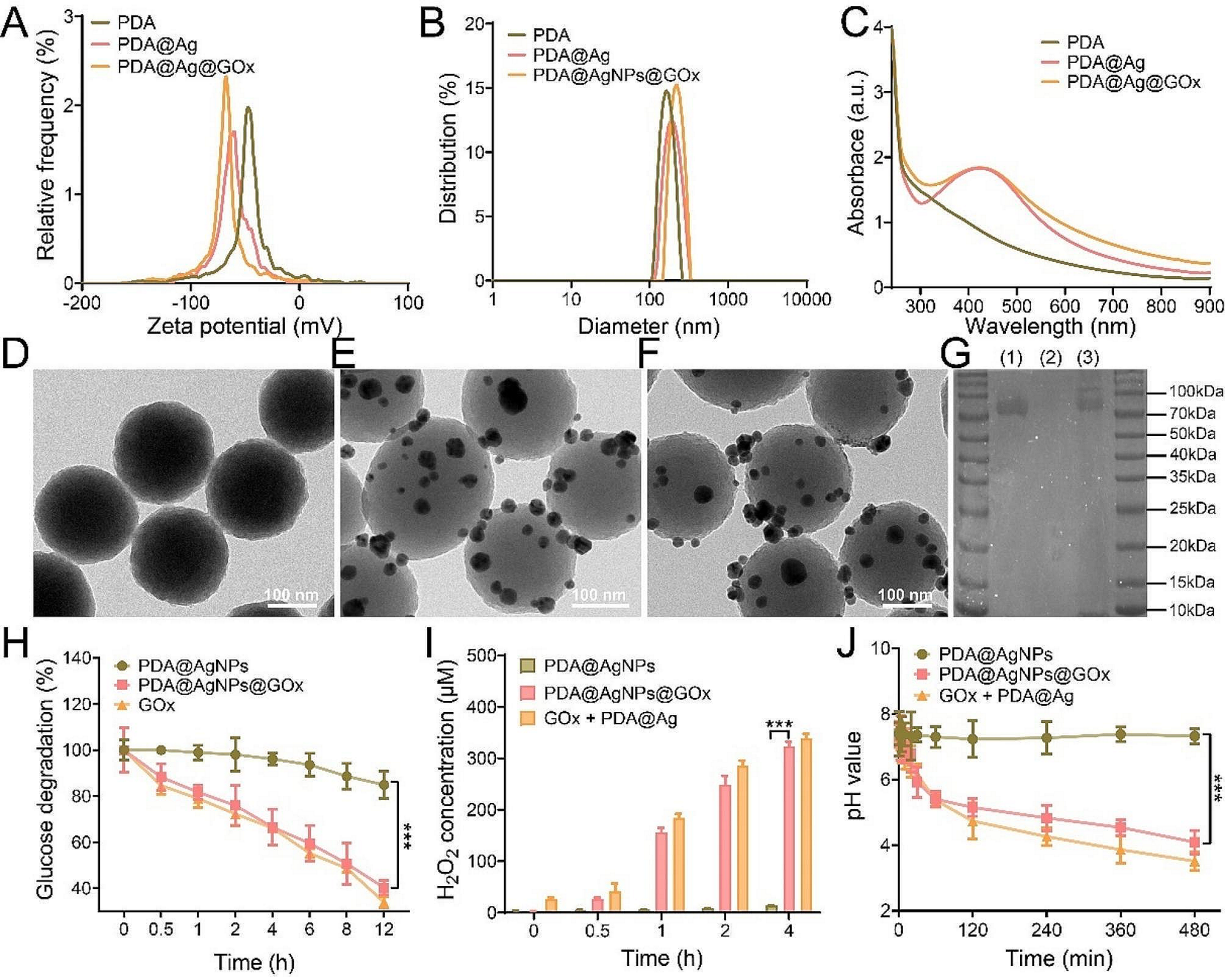
Characterization and performance of PDA@Ag@GOx. (**A**) Zeta potential and (**B**) hydrodynamic size distribution of PDA, PDA@Ag, and PDA@Ag@GOx. (**C**) UV absorption curves of PDA, PDA@Ag, and PDA@Ag@GOx at equal PDA concentration of 100 µg/mL. TEM images of (**D**) PDA, (**E**) PDA@Ag, and (**F**) PDA@Ag@GOx. (**G**) SDS-Page protein analysis of (1) GOx, (2) PDA@Ag, and (3) PDA@Ag@GOx. (**H**) Glucose degradation, (**I**) H_2_O_2_ generation, and (**J**) pH value at different times for different samples treated with glucose solutions (1 mg/mL) at equal GOx concentration (1.5 µg/mL). Significance was calculated via One-way ANOVA analysis

First, PDA was synthesized according to the classical method in the literature [25, 48]. Then, PDA@Ag was obtained by generating silver nanoparticles onto the PDA surface by in situ AgNO_3_ reduction [25, 49]. However, direct attachment of GOx to the surface of PDA@Ag was challenging due to their similar surface potential (both negatively charged) as illustrated in Fig. 1A and S1. To address this issue, the biocompatible positively charged PEI-600 was selected as an intermediate ligand to facilitate the loading of the negatively charged GOx via electrostatic adsorption [47, 50], thus obtaining the final nanomedicine PDA@Ag@GOx. Following the preparation of the materials, the morphology and size of the materials were observed. The size of the PDA@Ag@GOx particles was approximately 220 nm and exhibited uniform distribution, as depicted in Fig. 1B. More importantly, the hydrated particle size of PDA@Ag@GOx was larger than that of PDA@Ag (~ 180 nm) and PDA (~ 160 nm), which could laterally prove the successful loading of AgNPs and GOx to some extent. Meanwhile, it could be seen from Fig. 1A that the surface potential of PDA@Ag@GOx also changed compared with that of PDA, PDA@Ag. In order to visually and accurately confirm the successful loading of AgNPs and GOx, UV absorption, TEM and sodium dodecyl sulfate-polyacrylamide gel electrophoresis (SDS-Page) were utilized for analysis. As shown in Fig. 1C, the UV absorption curve of PDA@Ag and PDA@Ag@GOx exhibited a peak at approximately 420 nm, which is the characteristic absorption peak of AgNPs. Besides, the nanoparticle distribution of small particles could be observed in PDA@Ag and PDA@Ag@GOx, but not in PDA in TEM images (Fig. 1D-F). All the results confirmed the successful loading of AgNPs. As evidenced, the SDS-Page plot showed consistent protein bands of PDA@Ag@GOx with free GOx, indicating successful loading of GOx (Fig. 1G). Subsequently, the quantity of GOx in PDA@Ag@GOx was quantified using the BCA method, revealing a concentration of approximately 3% (Fig. S2). Furthermore, the stability of the nanoplatform under various physiological conditions was assessed by examining the long-term size distribution and surface potential of PDA@Ag@GOx in water, PBS, and DMEM over a period of 7 days. The hydrodynamic size and zeta potential of PDA@Ag@GOx remained relatively consistent across various storage media for 7 days, suggesting the stability of the material under diverse physiological conditions (Fig. S3).

The catalytic activity of PDA@Ag@GOx was further confirmed through protein fugacity. According to the literature, the catalytic ability of GOx is mainly manifested as the catalytic decomposition of glucose to produce H_2_O_2_ and gluconic acid [51], prompting an investigation into the corresponding properties. After adding PDA@Ag@GOx to the glucose solution (1 mg/mL), a decrease in glucose concentration was observed (Fig. 1H) using DNS method (Fig. S4), along with an increase in H_2_O_2_ concentration determined (Fig. 1I) by H_2_O_2_ assay kit (Fig. S5). Additionally, a decrease in pH value was noted, attributed to the production of gluconic acid as the reaction time increased (Fig. 1J). Furthermore, it is noteworthy that the glucose solution treated with PDA@Ag alone exhibited no substantial alterations in glucose concentration, H_2_O_2_ concentration, or pH value over time. Conversely, the observations consistent with the PDA@Ag@GOx group were evident in the PDA@Ag + GOx group. These findings collectively suggested that the catalytic activity of GOx remained unaffected throughout the synthesis of PDA@Ag@GOx.

### The enhanced PTT of PDA@Ag@GOx mediated by AgNPs decoration

**Fig. 2:**
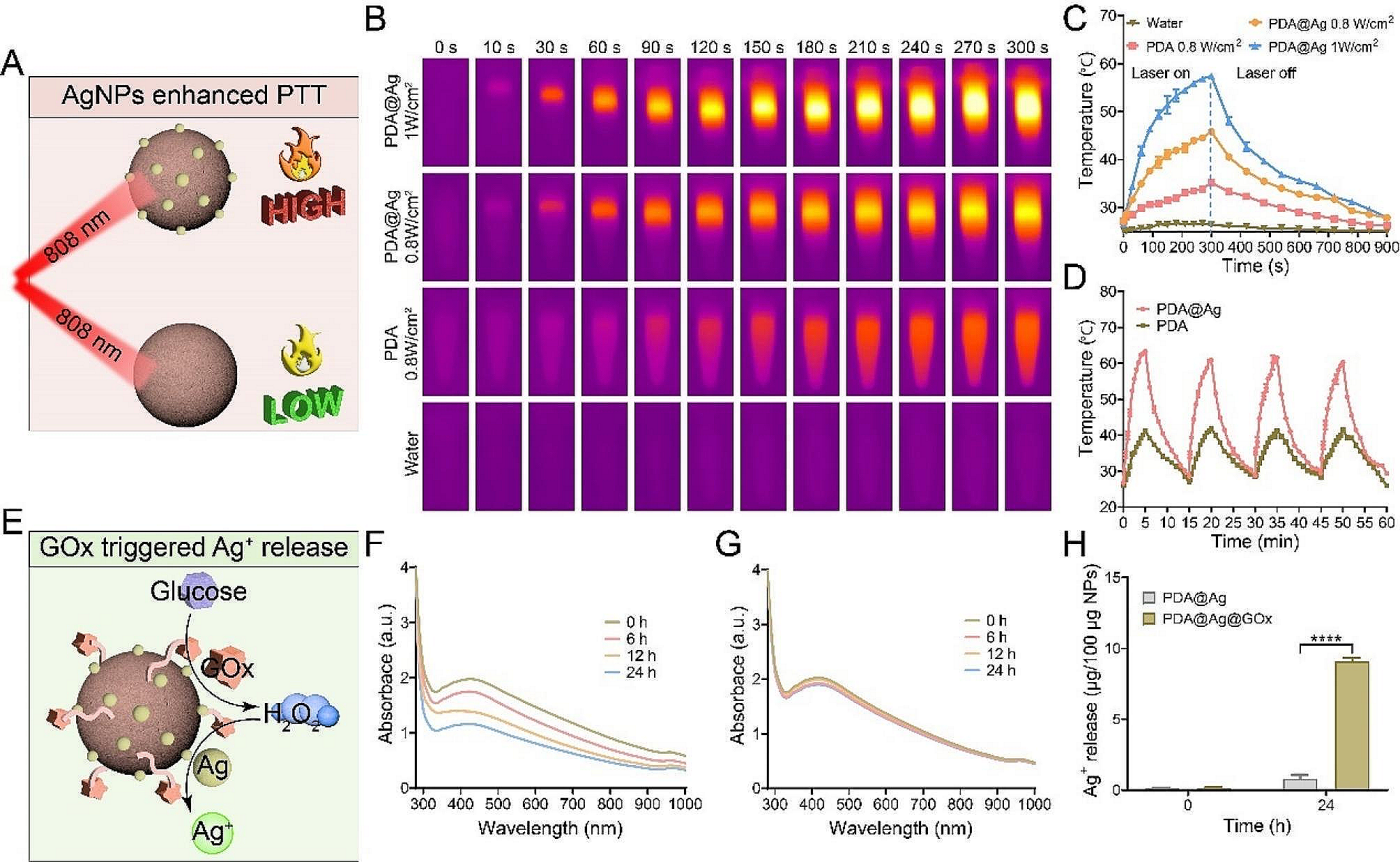
(**A**) Illustrative diagram of AgNPs-enhanced PTT. (**B**) Thermal images of PDA and PDA@Ag were recorded by an IR camera under laser irradiation (808 nm) with different power (0.8 W/cm^2^, 1 W/cm^2^) for 5 min, and (**C**) the corresponding heating curves. (**D**) Photothermal cycling curves of PDA and PDA@Ag under laser irradiation (808 nm, 1 W/cm^2^). (**E**) Schematic diagram of the process of GOx-triggered Ag^+^ release. UV absorption curves of (**F**) PDA@Ag@GOx and (**G**) PDA@Ag after incubation with glucose solution (1 mg/mL) at different times at equal PDA concentration of 150 µg/mL. (**H**) The Ag^+^ release of PDA@Ag@GOx and PDA@Ag after incubation with glucose solution for 24 h by ICP. Significance was calculated via One-way ANOVA analysis

Despite being a commonly utilized nano-photothermal agent, PDA is hindered by its limited photothermal conversion efficiency, resulting in inadequate PTT efficacy. Consequently, there is a need to enhance the photothermal therapeutic efficiency of PDA in order to generate heat more effectively at the tumor site for tumor ablation. According to the literature, the integration of PDA with metal nanoparticles can accelerate the charge transfer efficiency of PDA, which in turn enhances the non-radiative transition and ultimately improves the photothermal efficiency of PDA. Thus, we first verified the photothermal enhancement effect when PDA was decorated with AgNPs (Fig. 2A). From the liquid-phase thermographic images and the corresponding temperature quantification curves, it could be seen that the temperature of the PDA@Ag solution was higher than that of the PDA solution (with equal PDA concentration at 100 µg/mL) at the same irradiation power and irradiation time, indicating that the composite of AgNPs could indeed enhance the photothermal effect of PDA. In addition, the temperature of PDA@Ag solution was enhanced with the laser power at a consistent irradiation time, demonstrating that its photothermal efficiency can be conveniently controlled by adjusting the intensity of NIR light as well as the irradiation time (Fig. 2B and C). Following multiple laser irradiation cycles, the PDA@Ag solution exhibited a sustained maximum temperature without significant decrease, demonstrating its effective photothermal cycling capability (Fig. 2D). In addition, the photothermal conversion efficiency (η) of PDA and PDA@Ag was calculated to be 18.3% and 31.8% (Fig. S6), using the established calculation method from previous literature [52, 53], which further highlighted the enhanced photothermal effect of PDA through AgNPs modification (Fig. S7A and S7B). Furthermore, analysis of the photothermal images, photothermal curves, and photothermal conversion efficiency results (30.2%) of PDA@Ag@GOx (Fig. S7C) indicated that the loading of GOx had minimal impact on the photothermal effect of PDA@Ag. This suggested that PDA@Ag@GOx retained an exceptional photothermal conversion effect, which could be effectively utilized for tumor ablation through PTT.

### Toxic Ag^+^ release from PDA@Ag@GOx triggered by GOx

In addition to enhancing the photothermal efficiency of PDA, AgNPs are often used for antitumor and antibacterial purposes. However, the silver present in AgNPs typically exists in a minimally toxic 0-valent state, resulting in limited cytotoxic effects. However, AgNPs are highly susceptible to oxidation by ROS with strong oxidative activity and subsequent release of Ag^+^, which has a superior cell lethal ability compared to Ag^0^. Based on the theoretical analysis, we deduced that PDA@Ag@GOx could transform AgNPs from the inert state to the toxicogenic active state by catalyzing the production of H_2_O_2_ from glucose via GOx, thus achieving Ag^+^-mediated cell killing (Fig. 2E). Thus, the efficacy of PDA@Ag@GOx in inducing the release of Ag^+^ was initially confirmed through in vitro experiments. Considering that 420 nm is the specific UV absorption peak of AgNPs, PDA@Ag@GOx was immersed in solutions with or without glucose, and the variations in UV absorption were monitored over time. As depicted in Fig. 2F and G, the UV absorption peak at 420 nm for PDA@Ag@GOx in the glucose-containing solution exhibited a gradual reduction with prolonged soaking duration, whereas no significant changes were observed in the absence of glucose. Subsequently, the Ag^+^ content in the supernatant of PDA@Ag and PDA@Ag@GOx was analyzed after a 24 h immersion in a glucose solution using ICP analysis. It could be seen that a large amount of Ag^+^ was detected in the supernatant of PDA@Ag@GOx, in contrast to the minimal detection of Ag^+^ in the PDA@Ag solution (Fig. 2H). All the above findings demonstrated that the H_2_O_2_ generated by GOx-catalyzed glucose could indeed effectively trigger the release of Ag^+^ from AgNPs, providing a prerequisite basis for the subsequent Ag^+^-mediated cell killing effect.

### GOx-mediated PTT sensitization

**Fig. 3:**
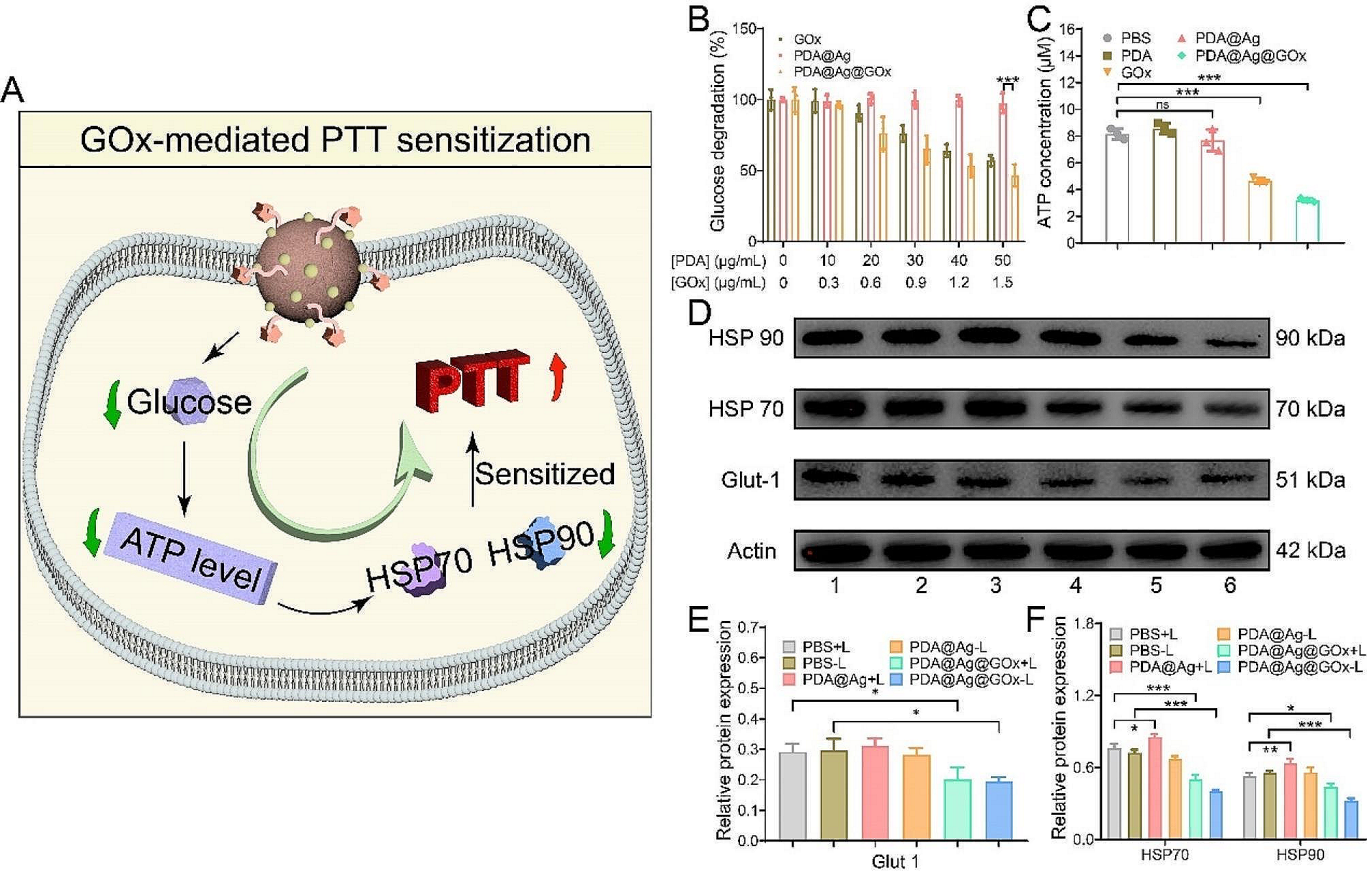
(**A**) Schematic diagram of the mechanism of GOx-sensitized PTT. (**B**) Glucose degradation at the cellular level after treatment with different concentrations of PDA@Ag@GOx, PDA@Ag, and GOx. (**C**) ATP levels in Hepa 1 − 6 cells after being treated with various samples at equal concentration of PDA (50 µg/mL) or GOx (1.5 µg/mL). (**D**) Western blotting analysis of Glut 1, HSP70, and HSP90 expression in Hepa 1 − 6 cells after various treatments at equal concentration of PDA (50 µg/mL) or GOx (1.5 µg/mL). (1) PBS + L, (2) PBS-L, (3) PDA@Ag + L, (4) PDA@Ag-L, (5) PDA@Ag@GOx + L, (6) PDA@Ag@GOx-L. The corresponding gray values of (**E**) Glut 1 and (**F**) HSP70 as well as HSP90. Significance was calculated via One-way ANOVA analysis

GOx not only triggers the release of Ag^+^ by catalyzing the production of H_2_O_2_ from glucose, but in the process, GOx interferes with cellular energy metabolism by consuming glucose and subsequently affects the synthesis of intracellular HSPs, thereby reducing the heat resistance of tumor cells and sensitizing the response to PTT (Fig. 3A). Subsequently, we conducted a systematic assessment of the capacity of GOx to enhance PTT. First, the ability of PDA@Ag@GOx to consume glucose at the cellular level was investigated. As shown in Fig. 3B, the intracellular glucose concentration showed a gradual decrease as the concentration of PDA@Ag@GOx increased, demonstrating its ability to consume glucose. Moreover, the glucose solution treated with PDA@Ag alone did not change significantly over time while that treated with GOx alone demonstrated a gradual decline in glucose levels. These outcomes suggested that GOx was primarily responsible for glucose depletion in the study. Subsequently, the intracellular ATP production capacity was investigated following various sample treatments. The ATP content detected in the cells treated with PDA@Ag@GOx was found to be significantly lower compared to the control group, which was similar to the group treated with GOx alone. Conversely, cells treated with PDA or PDA@Ag showed no significant reduction in ATP levels, suggesting that the presence of GOx hindered ATP synthesis in cells (Fig. 3C). This phenomenon was not only attributed to the direct depletion of glucose by GOx, but also because GOx downregulates glucose transporters 1 (Glut1), a major protein for glucose uptake by tumor cells [47, 54]. Glut1 expression was assessed in cells cultured in glucose-containing and glucose-free media, revealing a notable decrease in Glut1 expression in cells exposed to glucose-free conditions (Fig. S8). Furthermore, Glut1 expression was reduced in PDA@Ag@GOx-treated cells compared to untreated cells in the presence of glucose-containing medium (Fig. 3D and E). Conversely, no significant disparity in Glut1 expression was observed in PDA@Ag-treated cells compared to the control group. All these results indicated that GOx played a key role in down-regulating Glut1 to reduce cellular uptake of glucose, consistent with findings in the works of literature. After verifying the attenuation of intracellular energy metabolism, we finally investigated the synthesis of intracellular HSPs. From the western blot images and the corresponding semi-quantitative data, the levels of HSP70 and HSP90 were found to be elevated in cells treated with PDA@Ag and exposed to light irradiation compared to the control group. This increase can be attributed to the activation of the heat-resistant protection mechanism, which involves the upregulation of HSPs in response to external thermal stimulation. Conversely, the expression of HSPs was significantly downregulated in cells treated with PDA@Ag@GOx with or without light treatment, offering the possibility of sensitizing PTT by interfering with cellular energy metabolism (Fig. 3D and F). In order to further explore the interplay between GOx, HSPs, and energy levels within our system intuitively, we conducted experiments to measure ATP concentration and HSPs protein expression in different culture environments with and without the presence of both PDA@Ag@GOx and glucose, as depicted in Fig. S8. Cells cultured with glucose-free medium showed a significant decrease in intracellular ATP content as well as HSP70, HSP90 expression compared to cells cultured with glucose-containing medium. What’s more, when treated with PDA@Ag@GOx, the ATP and HSPs levels in cells were decreased regardless of the culture conditions with or without glucose, which further intuitively demonstrated the rationality of our proposal to sensitize PTT by regulating glucose metabolism and decreasing ATP synthesis, thus down-regulating HSPs.

### In vitro therapeutic effects of PDA@Ag@GOx

**Fig. 4:**
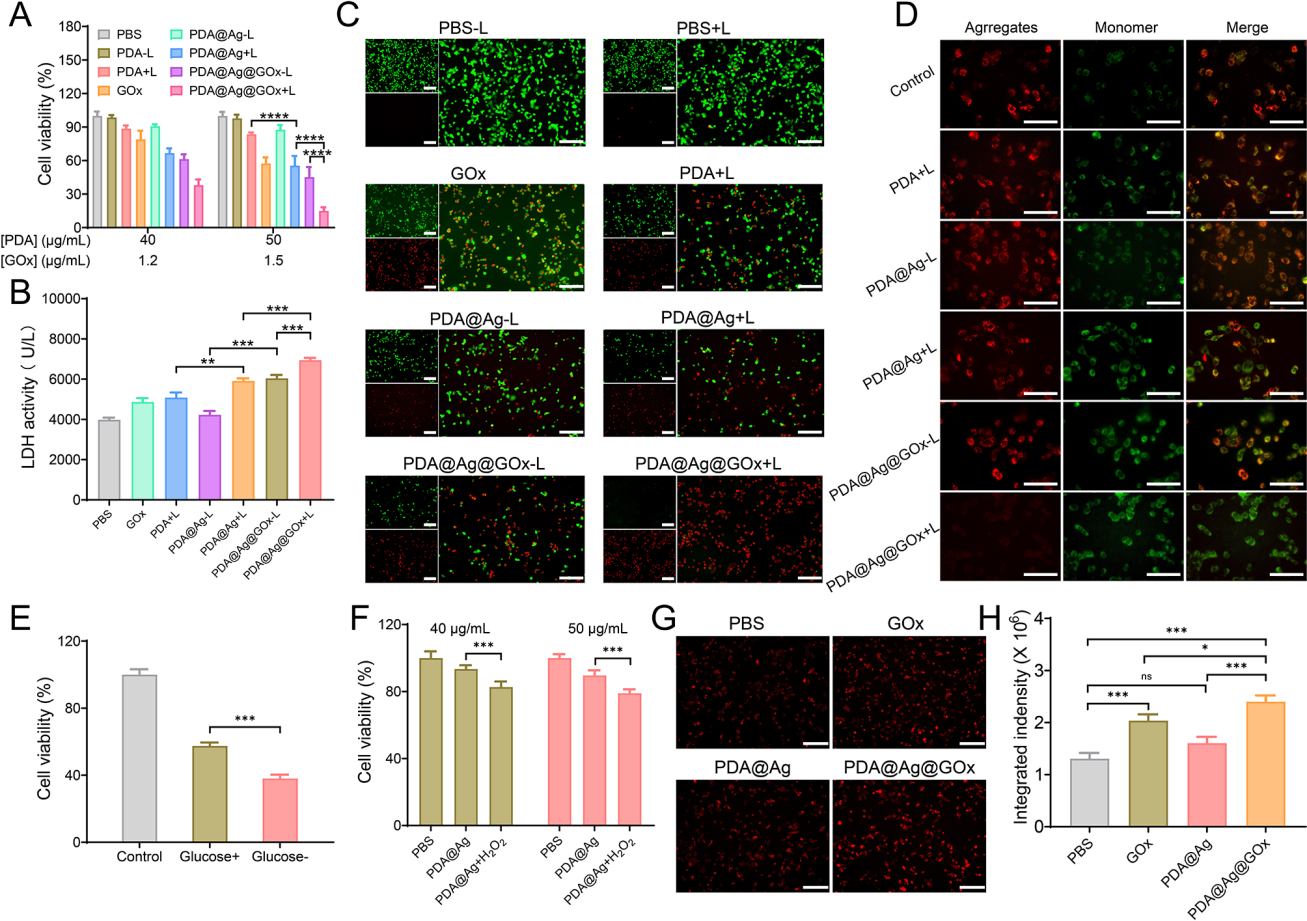
(**A**) The dark and light cytotoxicity of various samples against Hepa 1 − 6 cells by the MTT assay at equal concentration of PDA or GOx. (**B**) LDH activity detection of Hepa 1–6 cells after various sample treatments. (**C**) Live/dead cell staining images of Hepa 1–6 cells after various sample treatments. Scar bar is 100 nm. (**D**) JC-1 staining assay of Hepa 1–6 cells after different sample treatments. Scar bar is 100 nm. (**E**) The light cytotoxicity of PDA@Ag in the medium with or without glucose. (**F**) The dark cytotoxicity of PDA@Ag in the medium with or without H_2_O_2_. (**G**) The ROS detection of Hepa 1–6 cells after GOx, PDA@Ag, and PDA@Ag@GOx treatment using inverted fluorescence images at equal concentration of PDA (50 µg/mL) or GOx (1.5 µg/mL) and (**H**) corresponding semi-quantitative analysis. Scar bar is 100 nm. Significance was calculated via One-way ANOVA analysis

Having demonstrated the excellent photothermal enhancement, the toxic Ag^+^ release capacity, and the potential to sensitize PTT of PDA@Ag@GOx, its cytocidal effect against Hepa 1–6 cells was further investigated. As seen from the cytotoxicity results (Fig. 4A), the cells treated with PDA@Ag did not show cytotoxicity after dark treatment, indicating that AgNPs have not yet shown a killing effect on the cells. The survival rate of cells treated with PDA@Ag@GOx-L was lower compared to cells treated with equivalent concentrations of PDA@Ag or GOx, which was attributed to its catalytic production of H_2_O_2_ that activates the release of toxic Ag^+^, in addition to the toxic effect of GOx, thus enabling Ag^+^-mediated ion therapy (Fig. S9). In addition, the cell survival rate of PDA@Ag-treated cells was much smaller than that of PDA-treated cells at the same PDA concentration under light treatment, indicating that the enhanced photothermal effect due to AgNPs loading could indeed significantly enhance the cell killing effect. Most importantly, the PDA@Ag@GOx + L group exhibited the strongest toxic effect compared to PDA@Ag + L or PDA@Ag@GOx-L treated cells. This phenomenon was attributed to a “triple-linkage” effect between AgNPs-mediated enhanced photothermal effect, GOx-mediated Ag^+^ release, and GOx-sensitized cells in response to PTT. In addition, the disruption of cell membrane structure caused by cell death results in the release of lactate dehydrogenase (LDH) from the cell plasma into the culture medium [55]. By measuring the activity of LDH released into the culture medium from cells with ruptured plasma membranes, quantitative analysis of cytotoxicity can be achieved [56]. Consequently, we assessed the cytotoxic impact of nanotherapeutics on neoplastic cells by quantifying the amount of LDH discharged from cells subjected to varying treatments. As illustrated in Fig. 4B, the cells within the PDA@Ag@GOx + L group exhibited the highest release of LDH among all groups, consistent with the cytotoxicity findings, thus further confirming the remarkable anti-tumor efficacy of the nanoplatform. Subsequently, to visualize the cytocidal effect of PDA@Ag@GOx more thoroughly, live/dead cell staining experiments were performed immediately afterward. Expectedly, the strongest red fluorescence and the weakest green fluorescence expression were exhibited in cells treated with PDA@Ag@GOx and light, demonstrating its strongest cytocidal effect. In addition, a proportion of both red fluorescence and green fluorescence could be detected in PDA@Ag + L and PDA@Ag@GOx-L treated cells, while only strong green fluorescence could be detected in the control group (Fig. 4C). All these results were consistent with the above cytotoxicity results.

To elucidate the mechanism by which PDA@Ag@GOx induces apoptosis in tumor cells, we conducted JC-1 staining analysis. A reduction in mitochondrial membrane potential serves as a crucial indicator of apoptosis, prompting us to investigate alterations in mitochondrial membrane potential through JC-1 staining to validate the precise mechanism of cell death [57]. JC-1 emits red fluorescence when the mitochondrial membrane potential is high, while green fluorescence will be observed when the mitochondrial membrane potential is low. Therefore, when the ratio of green to red fluorescence is at a high level it represents a decrease in mitochondrial membrane potential, implying more apoptotic cell death. As shown in Fig. 4D, cells subjected to treatment with PDA@Ag@GOx and light exhibited the greatest green to red fluorescence ratio, indicating their superior efficacy in eliminating tumor cells through apoptosis.

To further explore the synergistic “triple-linkage” effect of PDA@Ag@GOx in tumor therapy, the distinct contributions of each component were assessed at the cellular level. Firstly, the phototoxicity of PDA@Ag was investigated in both glucose-containing and glucose-free medium. As shown in Fig. 4E, the phototoxicity of PDA@Ag in a glucose-free medium was greater than that in a glucose-containing medium, suggesting that reduced glucose levels did sensitize the cells in response to PTT. Additionally, the viability of cells treated with PDA@Ag in a medium containing H_2_O_2_ (500 µM) was approximately 75%, representing a decrease of approximately 17% compared to cells treated in a H_2_O_2_-free medium (92%). This suggested that H_2_O_2_ serves as a stimulus for the release of more toxic Ag^+^ ions from AgNPs, thereby augmenting the cytotoxic effect on cells (Fig. 4F). To circumvent the cytocidal effect of H_2_O_2_ itself, a concentration of H_2_O_2_ that was essentially non-cytotoxic was screened for the previous experiments (Fig. S10). This finding suggested that H_2_O_2_ did indeed activate the release of Ag^+^ from AgNPs and thus strengthen the cytotoxic effect.

It has been reported in the literature that Ag^+^-induced cell death is mostly attributed to its ability to cause cell peroxidation. Therefore, the ROS detection probe, DHE, was used to examine cells after different treatments [58, 59]. The results indicated that the red fluorescence observed in cells treated with PDA@Ag@GOx was more pronounced compared to cells treated with either PDA@Ag or GOx alone, suggesting an augmented toxic response due to the activation of Ag^+^ by GOx. At the same time, it has been previously demonstrated that PDA@Ag phototoxicity was stronger than the phototoxicity of PDA alone at equal concentrations of PDA (Fig. 4G and H). All these observations provided better validation that PDA@Ag@GOx achieved tumor cell killing through the interlocking effects of AgNPs-enhanced PTT, GOx-activated Ag^+^ release, and GOx-sensitized PTT action.

### In vivo photothermal conversion and imaging of PDA@Ag@GOx

**Fig. 5:**
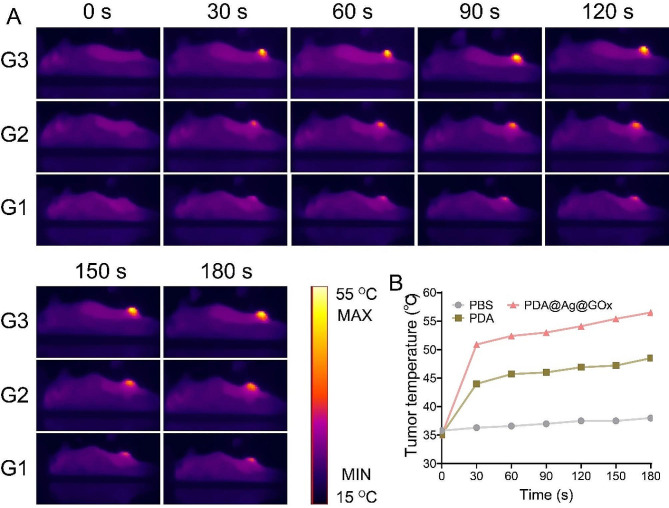
(**A**) In vivo photothermal images of mice after intravenous injection of PBS, PDA, and PDA@Ag@GOx. G1: PBS, G2: PDA, G3: PDA@Ag@GOx. (**B**) The corresponding temperature increase curve

In order to conduct a more comprehensive examination of the photothermal conversion efficacy of PDA@Ag@GOx in an in vivo setting, the alterations in temperature within tumor tissues were monitored through the utilization of infrared thermography under 808 nm laser exposure. From the thermal imaging photos and the corresponding temperature change curves (Fig. 5A and B), it could be seen that the temperature of the tumor tissues of mice injected with PDA@Ag@GOx via the tail vein gradually increased with the prolongation of the irradiation time, and was higher than that of mice injected with PDA at the same irradiation time. In addition, there was essentially no significant change in tumor temperature in mice treated with PBS only. These findings collectively indicated that the advantageous photothermal conversion capabilities of PDA@Ag@GOx in vivo are primarily due to the enhanced PTT effect of silver nanoparticles, rendering it a highly promising nano-formulation for achieving tumor thermal ablation.

### In vivo antitumor effect of PDA@Ag@GOx

**Fig. 6:**
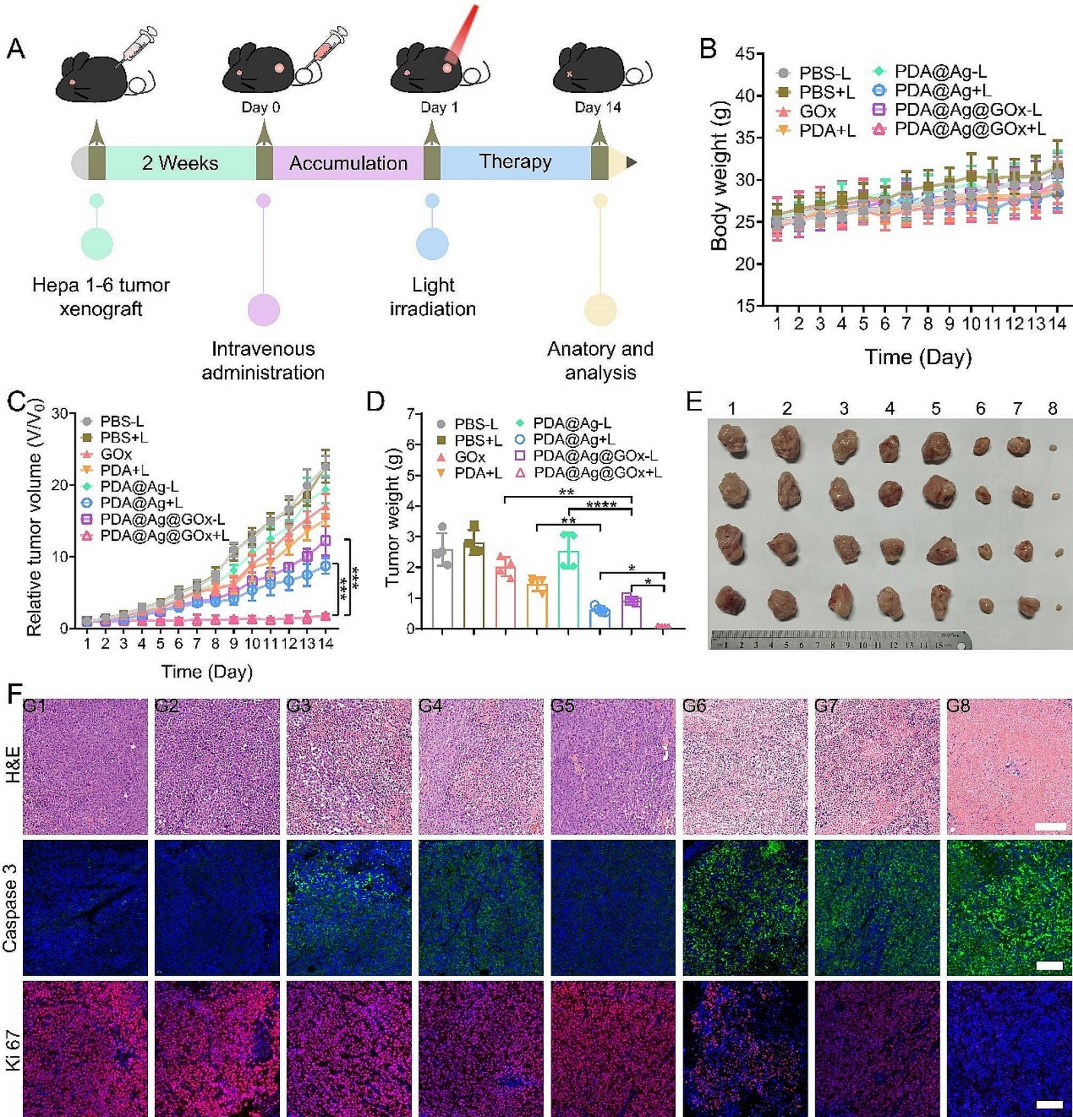
Antitumor efficiency of PDA@Ag@GOx in vivo. (**A**) Schematic illustration of the timeline for establishment and treatment of Hepa 1–6 tumor models. (**B**) The mice body weight and (**C**) the relative tumor volume changes of tumor-bearing mice in 14 days after various treatments at PDA concentration of 10 mg/kg mice body weight. (**D**) The average weights and (**E**) photographs of tumor tissues ex vivo were obtained on the 14th day. Significance was calculated via One-way ANOVA analysis. (**F**) H&E staining, Caspase 3, and Ki67 immunofluorescence staining of the tumor tissues after various treatments for 14 days. Scale car was 200 μm. G1: PBS-L, G2: PBS + L, G3: GOx, G4: PDA + L, G5: PDA@Ag-L, G6: PDA@Ag + L, G7: PDA@Ag@GOx-L, G8: PDA@Ag@GOx + L

Based on the proven tumor targeting as well as in vitro anti-tumor properties of PDA@Ag@GOx, its antineoplastic effect against Hepa 1–6 tumor-bearing mice was further validated in vivo. A tumor-bearing mice model was established by subcutaneously injecting Hepa 1–6 cells under the back skin of mice. When the tumor volume was about 100 mm^3^, the mice were randomly divided into 8 groups (6 mice/group), G1) PBS-L, G2) PBS + L, G3) GOx, G4) PDA + L, G5) PDA@Ag-L, G6) PDA@Ag + L, G7) PDA@Ag@GOx-L, G8) PDA@Ag@GOx + L (Fig. 6A). The effectiveness of the treatment is assessed by daily monitoring of the mouse weight and changes in tumor size. During the treatment period, the mice in each group showed a steady and slow increase in body weight (Fig. 6B), indicating the low systemic toxicity of the designed nanoplatform. As shown in Fig. 6C, the tumor volumes of the mice in the PBS-L and PBS + L groups changed consistently and were the fastest of all groups, indicating that the light conditions used in the experiments did not affect the growth of the tumors. Furthermore, the findings suggested that tumor growth rates were similar between the PDA@Ag-L and PBS groups, indicating that the presence of AgNPs in an inactive state did not hinder tumor growth inhibition. Conversely, the PDA@Ag@GOx-L group exhibited notably greater tumor suppression compared to the PDA@Ag-L and GOx-L groups, underscoring the role of GOx in activating AgNPs to release Ag^+^ that effectively target and kill tumor cells. Notably, the light-treated groups demonstrated enhanced tumor suppression in mice treated with PDA@Ag compared to those treated with PDA alone, with the PDA@Ag@GOx group showing the most pronounced suppression effect. This finding was attributed to the dual effect of the PTT effect of PDA enhanced by AgNPs and the PTT sensitized by GOx. At the end of 14 days of treatment, all mice were executed and tumor tissue was collected for photography and weighing. Among all groups, the tumor tissue of the PDA@Ag@GOx + L group was the smallest in size and lightest in mass, once again certifying its excellent anti-tumor capacity (Fig. 6D and E). Subsequently, the anti-tumor capacity at the cellular level of the nanoplatform was detected by H&E staining as well as Ki67 and caspase immunofluorescence staining. As seen in the H&E staining result, the PDA@Ag@GOx + L group had the highest number of dead tumor cells (Fig. 6F). At the same time, the PDA@Ag@GOx + L group expressed the strongest green fluorescence of caspase as well as the weakest red fluorescence of Ki67. Taken together, these results collectively demonstrated that PDA@Ag@GOx has excellent tumor elimination capacity under light conditions.

In addition to the notable therapeutic benefits, the assessment of in vivo biosafety of nanomedicines serves as a crucial parameter to be evaluated. Therefore, H&E staining of the major organs (heart, liver, spleen, lungs, and kidneys) was first performed at the end of the treatment. The results revealed that the structural integrity of the normal tissues in mice treated with the nanomedicine remained largely unchanged in comparison to the control group, suggesting the favorable biosafety profile of the employed nanomaterials (Fig. S11). Furthermore, it was observed in hemolysis assays that the treatment of erythrocytes with high concentrations of PDA@Ag@GOx did not cause erythrocyte rupture (Fig. S12). In addition, it was found that the liver function parameters (AST/AST) of mice injected intravenously with PDA@Ag@GOx did not differ significantly from those of normal mice (Fig. S13). These biosafety results demonstrate the safety of PDA@Ag@GOx in vivo and offer the possibility to realize its translational potential.

## Conclusion

In summary, we constructed a cascade-modulated nanoplatform, PDA@Ag@GOx, to realize dual-mode HCC therapy through enhanced PTT and activatable MIT. AgNPs have the potential to improve the photothermal conversion efficiency of PDA under NIR illumination through the enhancement of non-radiative transitions. Conversely, GOx interferes with tumor glucose metabolism, leading to decreased energy production and reduced synthesis of HSPs, thereby diminishing cancer resistance to hyperthermia and augmenting the efficacy of PTT. Furthermore, the H_2_O_2_ produced by GOx during glucose catalysis creates a highly oxidized intracellular environment capable of oxidizing inert AgNPs to release cytotoxic Ag^+^, facilitating cascade-activated Ag^+^-mediated MIT. Moreover, experiments related to biosafety indicated that the nano-platform exhibited favorable biosafety in vivo, characterized by low toxicity and minimal side effects. Ultimately, the nanosystem successfully facilitated the treatment of hepatocellular carcinoma by leveraging the cascade effect involving polydopamine, silver nanoparticles, and glucose oxidase. This cascade-regulated multimodal nanotherapeutic platform serves as a valuable model for the development of more effective tumor therapy systems.

### Electronic supplementary material

Below is the link to the electronic supplementary material.

Supplementary Material 1

## Data Availability

No datasets were generated or analysed during the current study.
