# Supplementary material for: A cascade nanosystem with “Triple-Linkage” effect for enhanced photothermal and activatable metal ion therapy for hepatocellular carcinoma
Source: J Nanobiotechnology. 2024 Jun 14;22:334. doi: 10.1186/s12951-024-02551-z (PMC11177488; doi:10.1186/s12951-024-02551-z)
Supplement: Supplementary file 1 — Supplementary Material 1 [file 12951_2024_2551_MOESM1_ESM.docx]

**A Cascade Nanosystem with "Triple-Linkage" Effect for Enhanced Photothermal and Activatable Metal Ion Therapy for Hepatocellular Carcinoma**

Shuo Yu^1#^, Huan Shen^2#^, Xi Chen^2^, Hong Wang^2^, Chenyang He^3^, Tinghua Hu^4^, Gang Cao^1^*, Lu Zhang^1,2^*

^1^Department of General Surgery, The Second Afﬁliated Hospital, Xi'an Jiaotong University, Xi'an 710000, China

^2^Department of Tumor and Immunology in Precision Medical Institute, The Second Afﬁliated Hospital, Xi'an Jiaotong University, Xi'an 710000, P. R. China

^3^The Breast Disease Diagnosis and Treatment Center, The Second Afﬁliated Hospital, Xi'an Jiaotong University, Xi'an 710000, P. R. China

^4^Department of Respiratory and Critical Care Medicine, The First Afﬁliated Hospital, Xi'an Jiaotong University, Xi'an 710000, P. R. China

Corresponding Author E-mail: paul5381@sina.com, Lulu-Zhang@xjtu.edu.cn


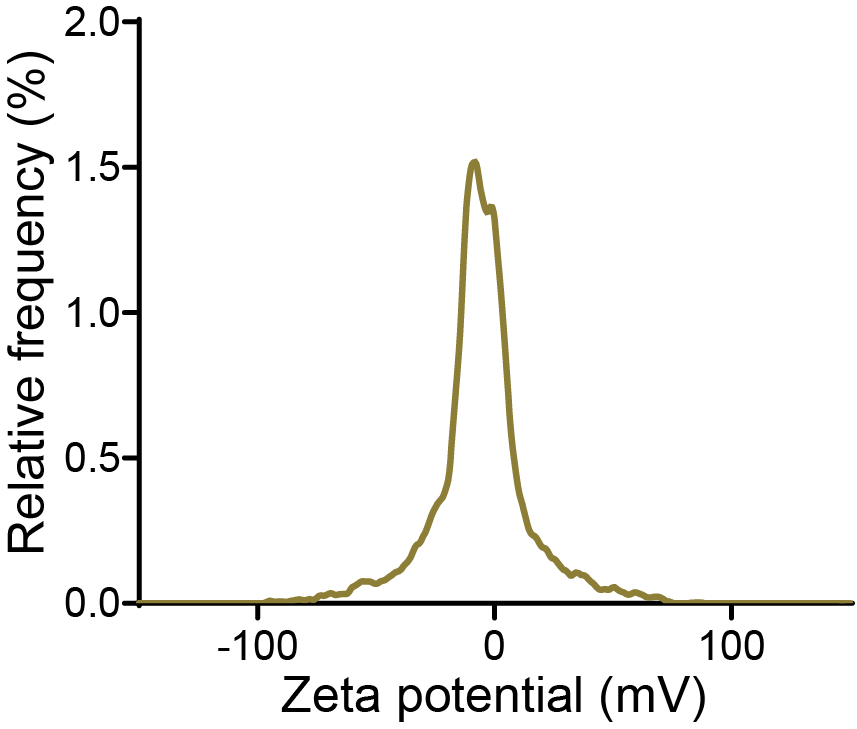


**Fig. S1** Zeta potential of GOx.


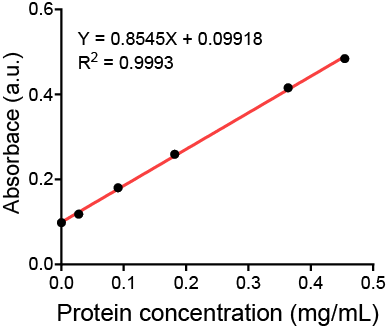


**Fig. S2** The standard curve of protein using BCA method. Y = 0. 8545X + 0.09918, R^2^ = 0.9993.


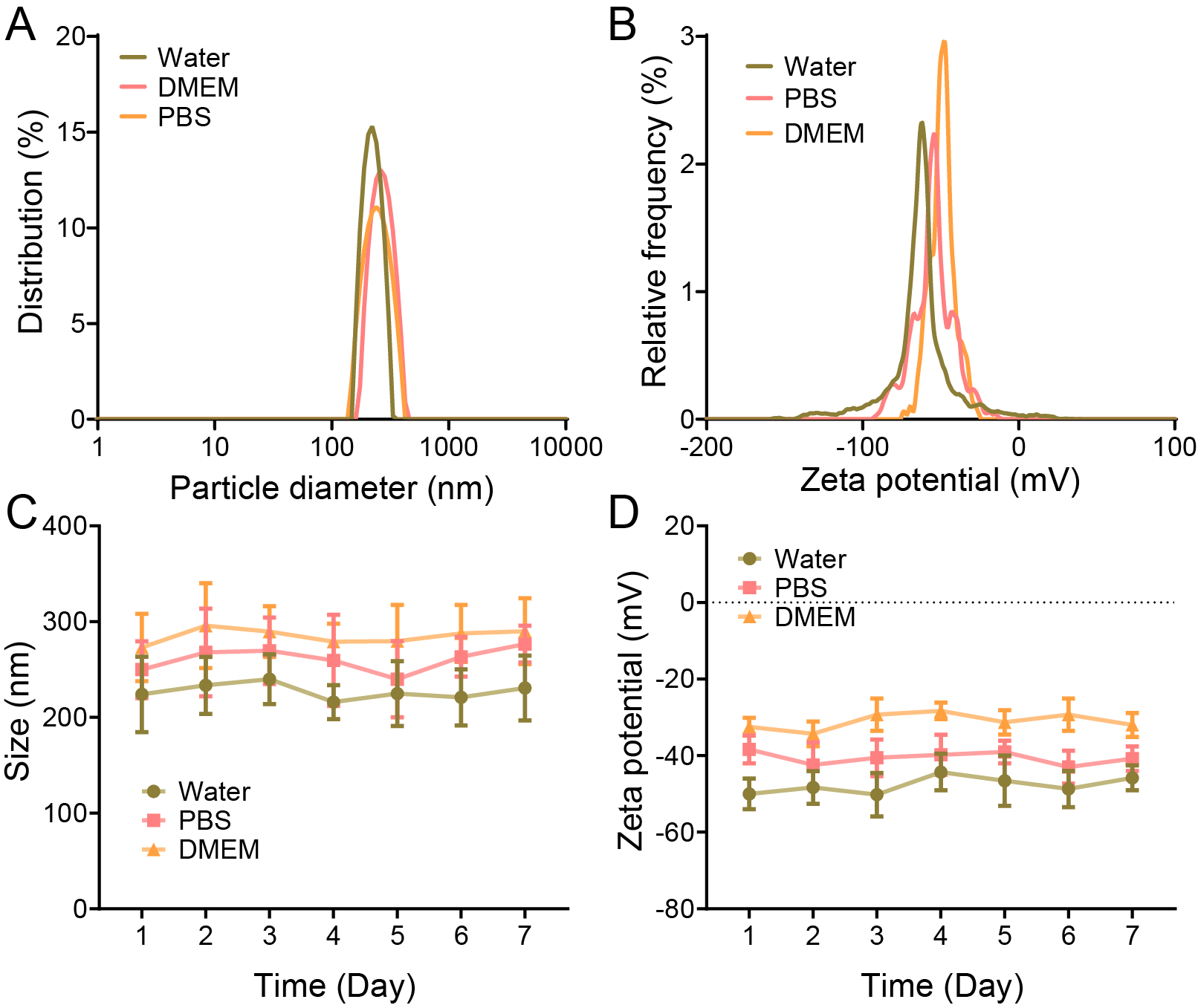


**Fig. S3** (A) Hydrodynamic size distribution and (B) zeta potential of PDA@Ag@GOx in water, PBS, and DMEM, respectively. (C) Hydrodynamic size and (D) zeta potential changes of PDA@Ag@GOx in 7 days.


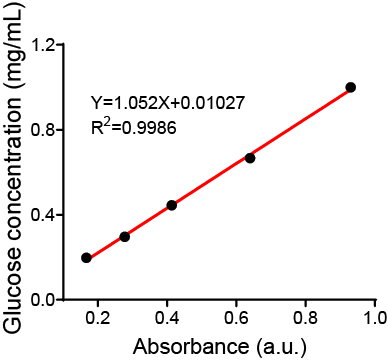


**Fig. S4** The standard curve of glucose using DNS method. Y = 1.052X + 0.01027, R^2^ = 0.9986.


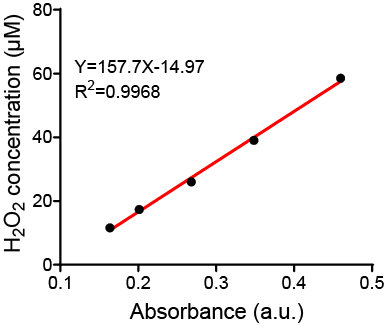


**Fig. S5** The standard curve of H_2_O_2_ using H_2_O_2_ detection kit. Y = 157.7X - 14.97, R^2^ = 0.9968.


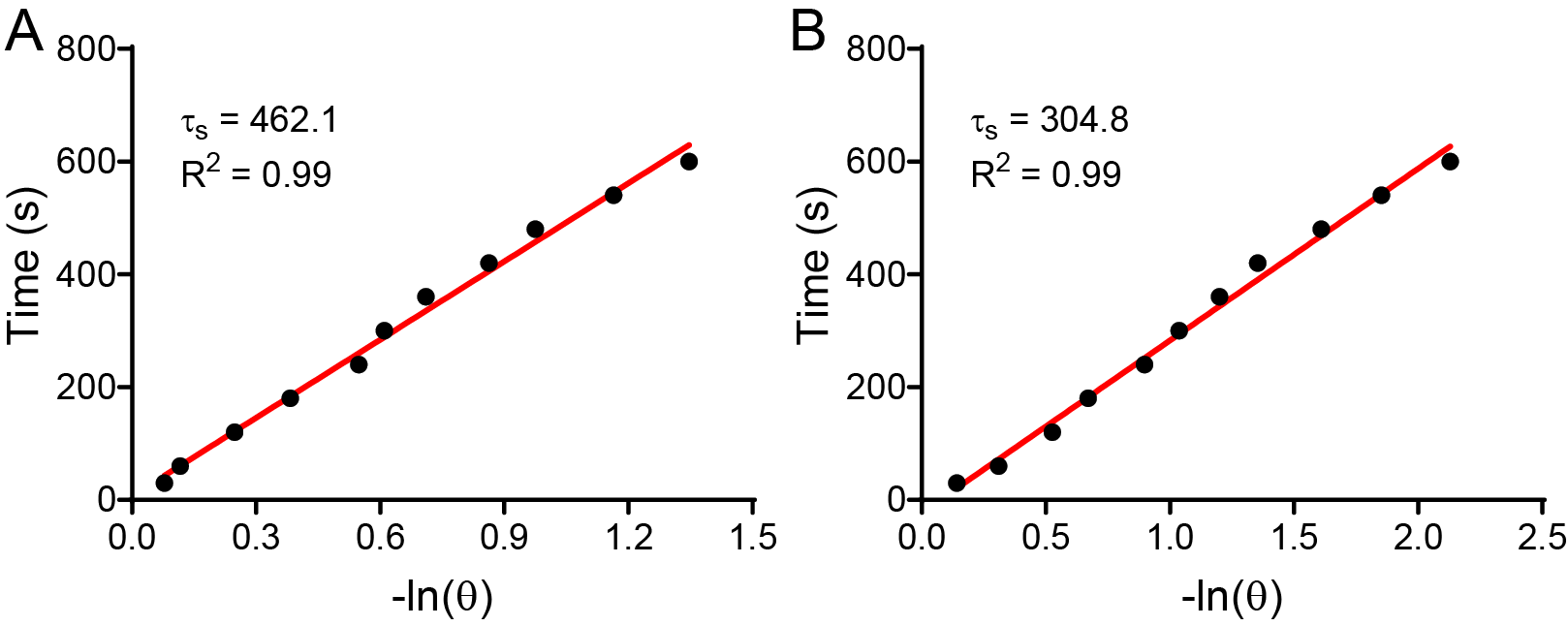


**Fig. S6** Linear fitting curve of the cooling time of (A) PDA, and (B) PDA@Ag as function of negative logarithm of temperatures (τ_s_ is the time constant of the sample system).


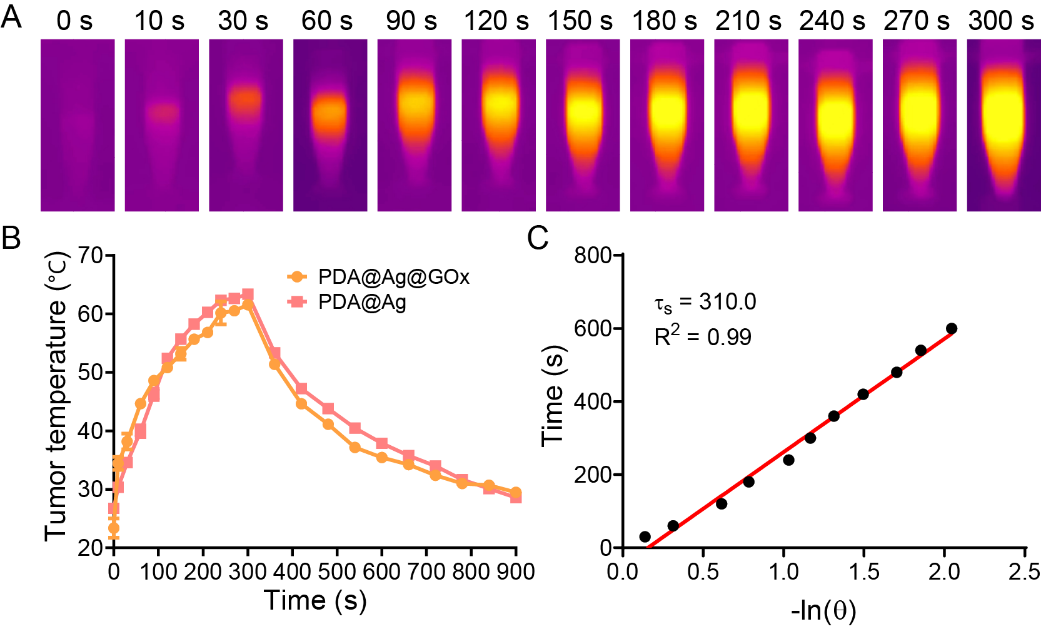


**Fig. S7** (A) Thermal images of PDA@Ag@GOx were recorded by an IR camera under laser irradiation (808 nm) with different power (1 W/cm^2^) for 5 min, and (B) the heating curves of PDA@Ag@GOx and PDA@Ag. (C) Linear fitting curve of the cooling time of PDA@Ag@GOx as a function of negative logarithm of temperatures (τ_s_ is the time constant of the sample system).


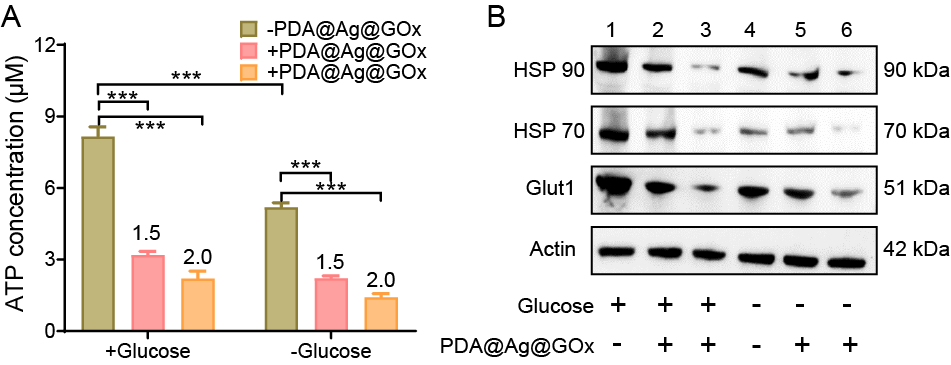


**Fig. S8** (A) ATP levels in Hepa 1−6 cells after being treated with DA@Ag@GOx (at GOx concentration of 1.5 or 2 μg/mL) in glucose-containing or glucose-free medium. Significance was calculated via Two-way ANOVA analysis. (B) Western blotting analysis of Glut 1, HSP70, and HSP90 expression in Hepa 1−6 cells in glucose containing or glucose-free medium with or without PDA@Ag@GOx treatment. 1,4: without treatment; 2, 5: treated with PDA@Ag@GOx at GOx concentration of 1.5 μg/mL; 3, 6: treated with PDA@Ag@GOx at GOx concentration of 2 μg/mL.


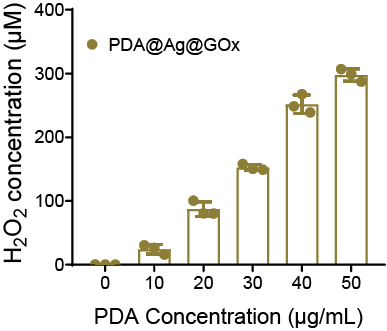


**Fig. S9** Intracellular H_2_O_2_ generation after treatment of Hepa 1-6 cells with different concentrations of PDA@Ag@GOx.


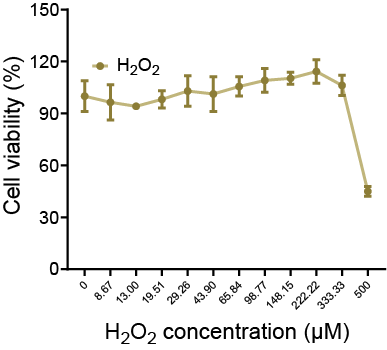


**Fig. S10** Cytotoxicity of different concentrations of H_2_O_2_ against Hepa 1-6 cells.


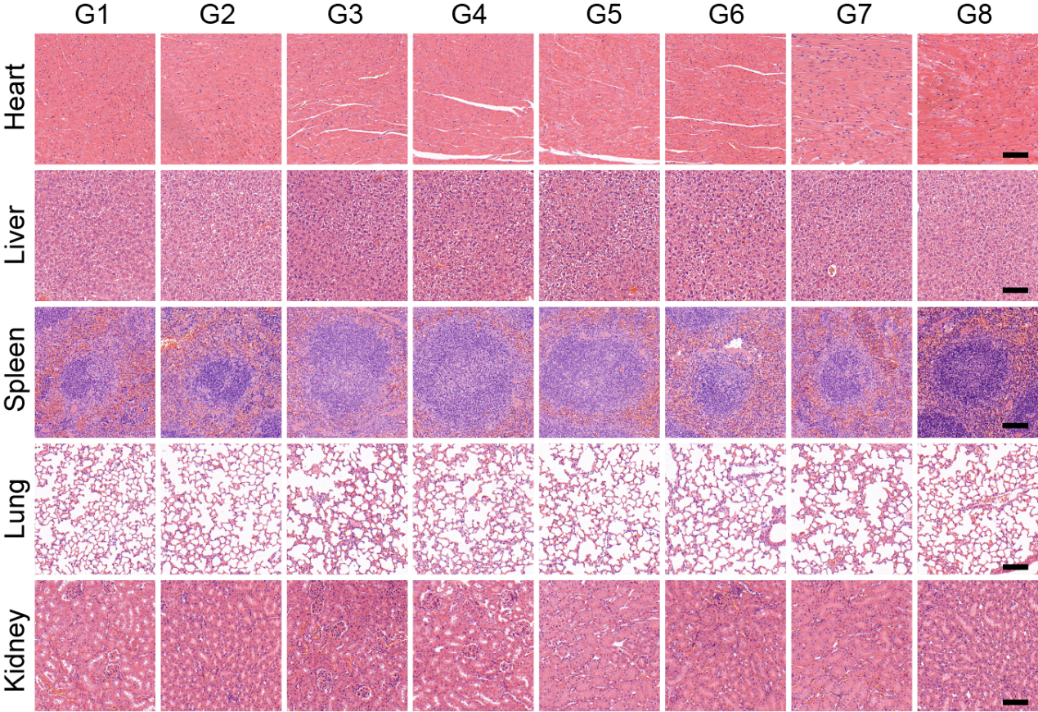


**Fig. S11** H&E staining of major organs of mice in different groups. G1: PBS-L, G2: PBS+L, G3: GOx, G4: PDA+L, G5: PDA@Ag-L, G6: PDA@Ag+L, G7: PDA@Ag@GOx-L, G8: PDA@Ag@GOx+L. Scale bar: 100 μm.


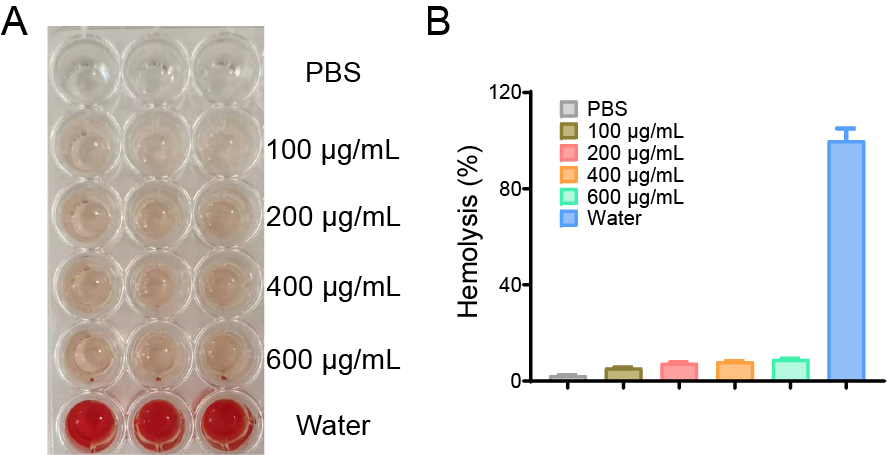


**Fig. S12** Hemolysis assay of PDA@Ag@GOx.


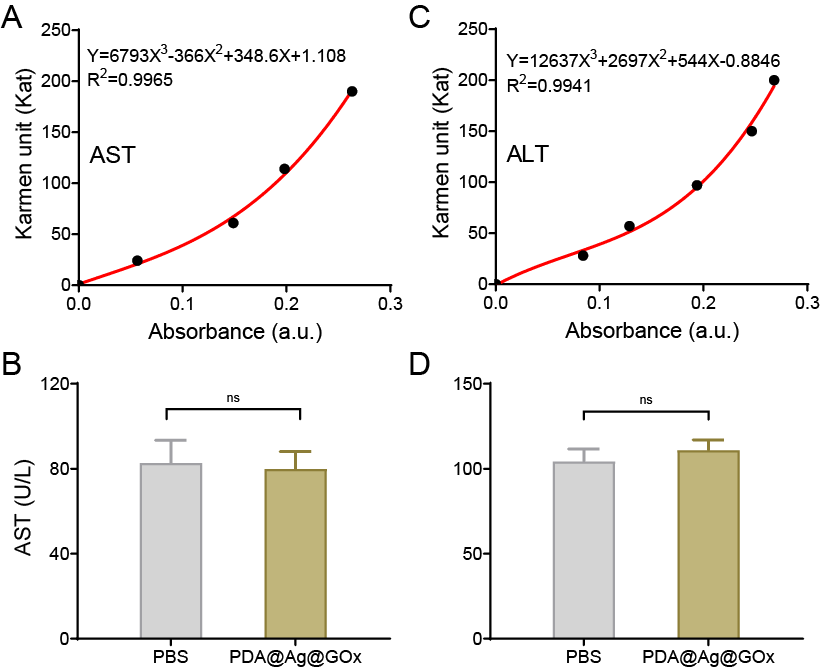


**Fig. S13** The ALT and AST levels of mice after treated with PBS and PDA@Ag@GOx, respectively.
